# Identification of Bacterial Composition in Freeze-Dried *Agaricus bisporus* During Storage and the Resultant Odor Deterioration

**DOI:** 10.3389/fmicb.2019.00349

**Published:** 2019-02-26

**Authors:** Wenjian Yang, Liuqing Wang, Qiuhui Hu, Fei Pei, Mariga Alfred Mugambi

**Affiliations:** ^1^Key Laboratory of Grains and Oils Quality Control and Processing, Collaborative Innovation Center for Modern Grain Circulation and Safety, College of Food Science and Engineering, Nanjing University of Finance and Economics, Nanjing, China; ^2^Faculty of Agriculture and Food Science, Meru University of Science and Technology, Meru, Kenya

**Keywords:** *Agaricus bisporus*, water activity, volatile compounds, metagenomics, bacterial community, bacterial diversity

## Abstract

Moisture absorption and bacterial growth are critical factors for quality deterioration of freeze-dried *Agaricus bisporus.* In order to explore the bacterial composition and the resultant odor changes in freeze-dried *A. bisporus* during storage under three typical conditions (RT: 25°C, 55% RH; HT: 37°C, 85% RH; AT: ambient temperature), bacterial diversity and communities were analyzed using metagenomics. Moreover, volatile compounds were determined using SPME-GC-MS. The results demonstrated that the bacterial composition in freeze-dried *A. bisporus* was dominated by *Pseudomonas*, followed by *Rhizobium* and *Pedobacter*. In addition, *Mucilaginibacter*, *Flavobacterium*, and *Thermus* were a few other genera more dominant in HT samples, *Chryseobacterium* was the other genera more dominant in AT samples, while, *Sphingobacterium* and *Chryseobacterium* were a few other genera more dominant in RT samples. Furthermore, the increase of benzaldehyde content in HT samples may have been induced by the growth of Pseudomonads and the esters production in RT and AT samples might have been induced by *Chryseobacterium*. This study provided comprehensive information on exogenous bacterial composition and the resultant odor in freeze-dried *A. bisporus.* These results may be a theoretical basis for quality control and quick quality detection based on volatiles of freeze-dried *A. bisporus*.

## Introduction

*Agaricus bisporus* are popular and valuable foods, which are low in calories but higher in minerals, protein, and fiber (Wang et al., 2014). *A. bisporus* tend to be traded as dried products in the market owing to short shelf-life (Ren et al., 2014). Freeze-dried *A. bisporus* have received much attention for their good sensory quality and high level of nutrient retention (Wang et al., 2015). Our previous study proved that the moisture content of freeze-dried *A. bisporus* continuously increased during the storage period of 25 days. The water absorption resulted in the quality deterioration, such as color, texture, nutrient and flavor (Wang et al., 2018). Moreover, bacterial growth, influenced by water, might result in spoilage and quality deterioration in freeze-dried *A. bisporus* (Ong et al., 2002). However, the effect of bacterial growth on the flavor deterioration of freeze-dried *A. bisporus* needs to be further discussed.

The low water activity of dried foods could effectively control the growth of foodborne pathogens. Likewise, the increasing water activity during storage accelerates the bacterial growth of the freeze-dried *A. bisporus* (Beuchat et al., 2013). Water activity represents water available for microbial spoilage in food and is often used to examine if the food has reached the critical scale at which spoilage reactions may occur or not (Mathlouthi, 2001). Moreover, environmental factors including moisture availability and temperature effect the bacterial diversity and composition. This may result in different quality changes of freeze-dried *A. bisporus* (Alvarez-Ordonez et al., 2015; Fogele et al., 2018). Thus, systematic analysis of overall bacterial diversity and community composition is significant in quality control of freeze-dried *A. bisporus* during storage.

Some studies reported that the bacterial growth in food products resulted in various kinds of metabolites including volatile compounds (Jaffres et al., 2011; Tait et al., 2014; De Vrieze et al., 2015). Joffraud et al. (2001) reported that *B. thermosphacta* in cold-smoked salmon were responsible for the production of 2-hexanone and 2-heptanone shown as sour and pungent off-flavor. Hernandez-Macedo et al. (2012) demonstrated that butanoic acid, 1-butanol can serve as a marker for spoilage species associated with blown pack vacuum chilled meat. Overall, these studies indicated a potential importance of volatile compounds as markers in bacterial contamination detection. Consequently, understanding the correlation between bacterial composition and volatiles is essential for the quality prevention and detection of freeze-dried *A. bisporus* during storage. However, to the best of our knowledge, the relationship between volatile compounds and bacterial composition in freeze-dried *A. bisporus* remains unexplored. Therefore, this work may provide useful reference information for development of safety control and quality detection of dried products.

The objective of this study was to identify some dominant bacteria and volatile markers in freeze-dried *A. bisporus* by profiling the bacterial composition and volatile compounds changes during storage. This might provide fundamental information for quality control and quick quality monitor of freeze-dried *A. bisporus*.

## Materials and Methods

### Sample Preparation

Freeze-dried *A. bisporus* slices were produced according to our previous method (Wang et al., 2018). After stems being removed, fresh *A. bisporus* were sliced to a thickness of 5 mm and distributed uniformly on a tray, then pre-frozen in −80°C for over 8 h. Then samples were lyophilized with a freeze dryer (Labconco Equipment Co., Kansas City, MO, United States) until moisture content is less than 5%. The freeze-dried *A. bisporus* slices were randomly packaged in normal polyethylene (PE) packing bag (20 g/bag) and, respectively, stored in three conditions for 25 days to simulate the storage condition of dried food: constant room temperature and humidity (25°C, 55% RH; named as RT), constant high temperature and high humidity (37°C, 85% RH; named as HT), variable ambient temperature and humidity (12–28°C, 50–90% RH; named as AT). Water activity, total viable counts, Pseudomonads and volatile compounds of freeze-dried *A. bisporus* under different conditions were determined every 5 days during storage.

### Water Activity Measurements

Water activity of the samples was determined with an a_w_ meter (Novasina AG, Lachen, Switzerland) (Dagnas et al., 2017), and the average of 10 replicates of each sample was recorded.

### Microbiological Analysis

Microbial changes (total viable counts and Pseudomonads) in freeze-dried *A. bisporus* under three storage conditions were monitored. Samples were homogenized (1:10) with phosphate buffer solution (10 mmol/L, pH = 7.2) in the stomacher bag, and mixed using a Stomacher 400 (International P. B. I., Milano, Italy) at 300 rpm for 2 min. The homogenate was gradiently diluted in phosphate buffer solution (10 mmol/L, pH = 7.2) for the following tests. Total viable counts were determined on plate count agar (PCA) and incubated at 36 ± 1°C for 48 h ± 2 h (Xiao et al., 2013). Pseudomonads colony counts were determined using cetrimide–fucidin–cephaloridine agar and incubated at 25 ± 1°C for 44 h ± 4 h (Balamatsia et al., 2006). Average of repeated determinations was translated into the number of colony forming unit (CFUs/g).

### DNA Extraction and Sequencing Libraries Construction

Samples on day 0 (CK samples) and samples stored for 25 days at RT, HT, and AT (RT, HT, and AT samples) were chosen for 16S rDNA sequencing analysis. Total DNA was extracted from bacterium solution by the TIANamp Bacteria DNA kit (TIANGEN Biotech, Beijing, China) following the manufacturer’s instruction (Mulla et al., 2018). The V4 region primer set was chosen to obtain the best coverage of most environmental microorganisms (Tyx et al., 2016). On the other hand, the universal primers 515F and 806R were used to amplify the V4 hypervariable regions of the 16S rDNA gene of bacteria (Herlemann et al., 2011).

Forward primer: 515F GTGCCAGCMGCCGCGGTAA;

Reverse Primer: 806R GGACTACHVGGGTWTCTAAT.

The PCR program included one denaturing step at 94°C for 2 min, 25 cycles of 94°C for 20 s, 55°C for 30 s, and 72°C for 1 min, followed by a final extension at 72°C for 10 min and 4°C forever. Then, the 16S rDNA from samples were examined with MiSeq sequencing by Genesky Biotechnology Inc. (Shanghai, China). High-throughput sequencing was performed on the Illumina Miseq platform with the 2 × 250 bp paired-end method after the library was quantified. To identify the OTUs presented in these samples, average linkage hierarchical clustering was used to cluster the partial 16S rDNA sequences at 97% sequence identity (Edgar, 2013; Wang et al., 2016). After quality cleaning, filtering and dereplication, the input sequences were ordered according to their abundances, considering the high abundance reads. Mothur was utilized for taxonomical assignments at 80% confidence level based on the Ribosomal Database Project (RDP) database (Wang et al., 2007; Luo et al., 2013). The main steps in the sequence filtering were: (1) selection of the sequences which contained barcode and forward primer and eliminate sequences with even a single base pair, (2) removal of sequences shorter than 150 bp, with ambiguous base pairs, or with more than two wrong matches in primer, (3) elimination of barcodes and forward primers. Then the effective sequences were clustered to OTUs based on phylum, family, genus and species levels using the MOTHUR program. RDP utilizes the weighted neighbor-joining algorithm of phylogenetic reconstruction. Weighbor parameters were set to alphabet size 4 and effective sequence length 1,000, and the Jukes-Cantor distance correction was employed. R software packages were employed for calculation of α- and β-diversities. Cluster analysis was performed by using the Bray–Curtis dissimilarity index and the unweighted pair-group method with arithmetic means (UPGMA) linkage method. The difference in bacterial communities of these groups was revealed by linear discriminative analysis (LDA) effect size (LEfSe) tests. The heatmap plot depicts the relative abundance of each bacterial family (variables clustering on the *Y*-axis) within each sample (*X*-axis clustering) (Xie et al., 2017). Metastats was used to identify which members within a community were responsible for differences between communities (White et al., 2009). The sequence data have been deposited in the National Center for Biotechnology Information (NCBI) Sequence Read Archive (SRA) under the accession number in PRJNA511927.

### HS-SPME-GC-MS Analysis

Volatiles were extracted by headspace solid phase microextraction (HS-SPME). Freeze-dried *A. bisporus* powder (0.5 g) were weighed in a 20 ml vial with 1 μL internal standard of 1-decanol methanol solution (80 mg/mL) (Li et al., 2011). The fiber holder (DVB/CAR/PDMS, 50/30 μm) (Supelco Ltd., Bellefonte, PA, United States) was used to extract the volatile compounds in a 60°C water bath for 45 min. GC-MS analysis was performed on Agilent 7890A/5975C GC-MS instrument (Agilent Technologies Inc., Santa Clara, CA, United States) according to reported method (Pei et al., 2016). The analytes were finally desorbed for 5 min at 250°C in the GC injector in splitless mode, then separated on a DB-5MS capillary column (30 m × 0.25 mm, 0.25 mm) (J&W Scientific, Folsom, CA, United States). Column temperature was initially maintained at 40°C for 3 min, then increased to 80°C at 5°C/min and held for 3 min, and raised to 220°C at 10°C/min for 2 min and finally to 240°C at 5°C/min for 2 min. The carrier gas was helium at a flow rate of 0.8 mL/min. Mass spectra was obtained in an electron impact mode. MS was taken at 70 eV ionization energy in the 35–550 amu mass range, with the ion source temperature at 230°C. The volatile compounds were tentatively identified by matching the mass spectra with the spectra of reference compounds in both the Wiley mass spectra library (sixth edition) and the NIST/EPA/NIH mass spectra library (version 1.5a). The results from volatile analyses are provided in peak area counts of the compounds identified. All experiments were performed in triplicate.

### Statistical Analysis

The experimental data from the HP-SPME-GC-MS was analyzed using the statistical software, PASW statistic 18. The data are expressed as the mean ± standard deviation (SD). The measured data were analyzed by SAS system, Version 9.0 (SAS Institute, Cary, NC, United States). Least significant differences (LSD) multiple comparison tests were then performed with a 95% confidence level.

## Results

### Water Activity and Microbiological Analysis

**FIGURE 1:**
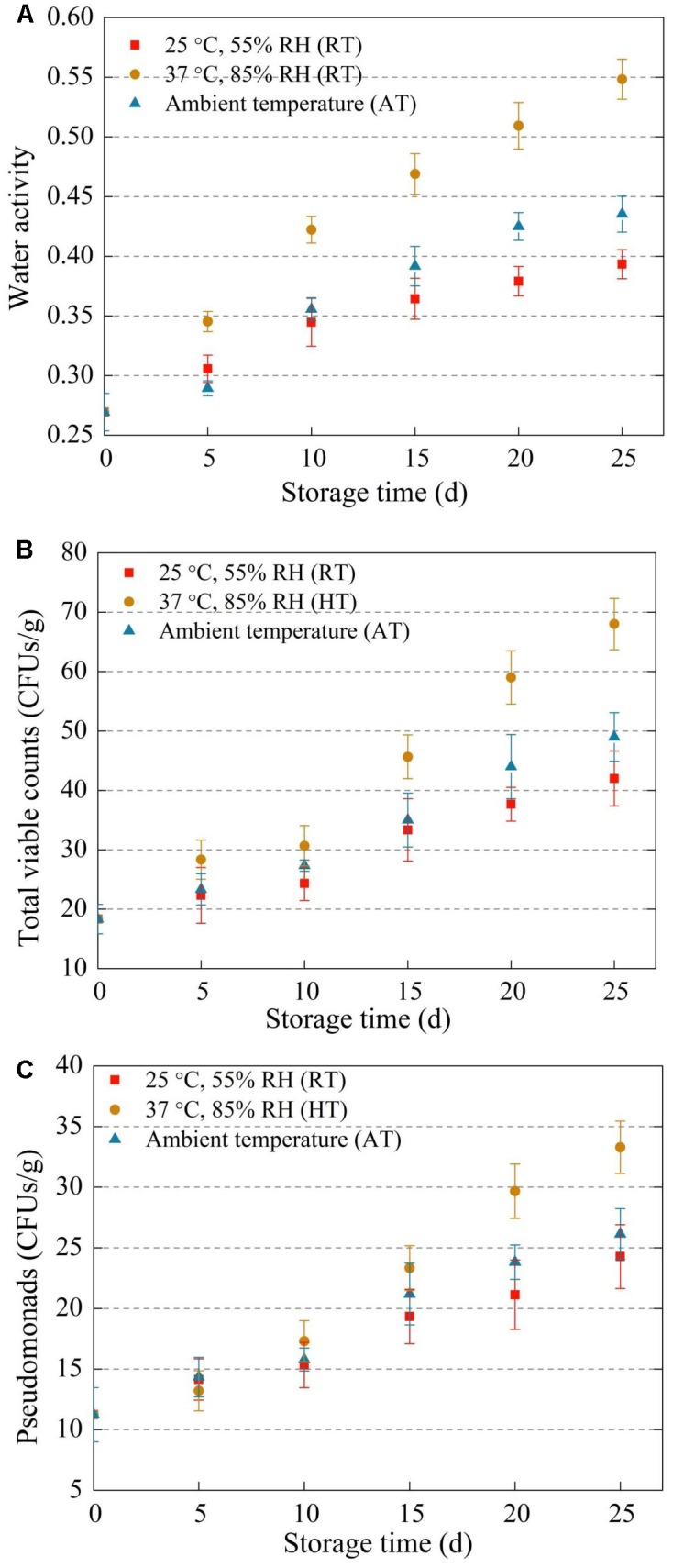
Water activity **(A)**, total viable counts **(B)**, and Pseudomonads **(C)** in freeze-dried *Agaricus bisporus* under different storage conditions.

Water activity is a vital parameter in dried foods quality and stability. Water activity, total viable counts, and Pseudomonads of freeze-dried *A. bisporus* in the three storage conditions were shown in Figure 1. Water activity, total viable counts and Pseudomonads all increased during storage. Water activity of HT samples was higher than that of AT and RT samples. Moreover, total viable counts and Pseudomonads colony counts of HT samples were significant higher than two other groups after day 15. In addition, total bacteria colony counts had significant positive correlation with water activity in the whole storage time (*p* < 0.05).

### Alpha Diversity of Bacteria

**FIGURE 2:**
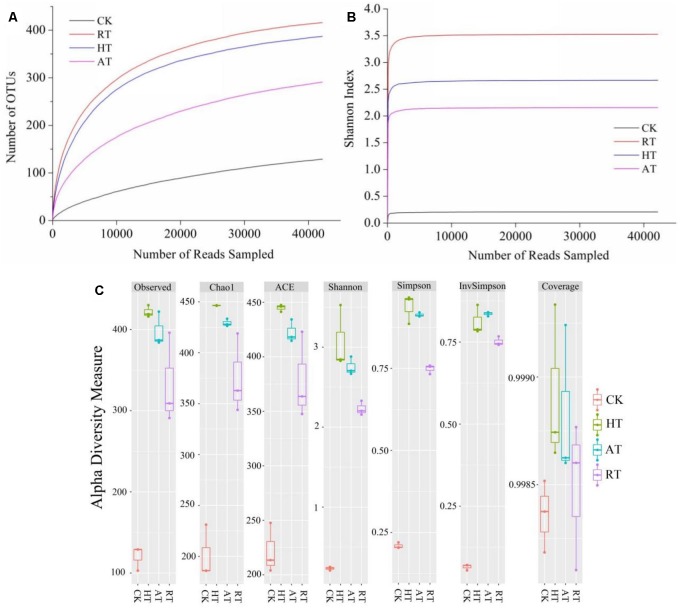
Rarefaction curve **(A)**, Shannon index curves **(B)**, and Alpha diversity **(C)** of freeze-dried *Agaricus bisporus* under different storage conditions.

Sequencing of 16S rDNA genes from the exogenous bacteria of freeze-dried *A. bisporus* was identified as 837 OTUs. Rarefaction curves, Shannon index curves and alpha diversity index were shown in Figure 2. The results showed that rarefaction curves evaluating the OTU richness approached saturation, indicating that the sampling covered almost the full taxonomic diversity at these genetic distances. Alpha diversity measure analysis showed observed, Chao1, ACE, Shannon, Simpson, InvSimpon and Coverage indices on the basis of the OTU number calculated to determine species richness and diversity. Among these groups, CK samples exhibited the lowest diversity while HT samples had the highest. Across all measures of alpha diversity, except Coverage, diversity of the HT samples had the highest species richness, followed closely by diversity of AT and RT samples. Thus, the diversity of bacterial community significantly increased in three storage conditions.

### Beta Diversity Analysis

**FIGURE 3:**
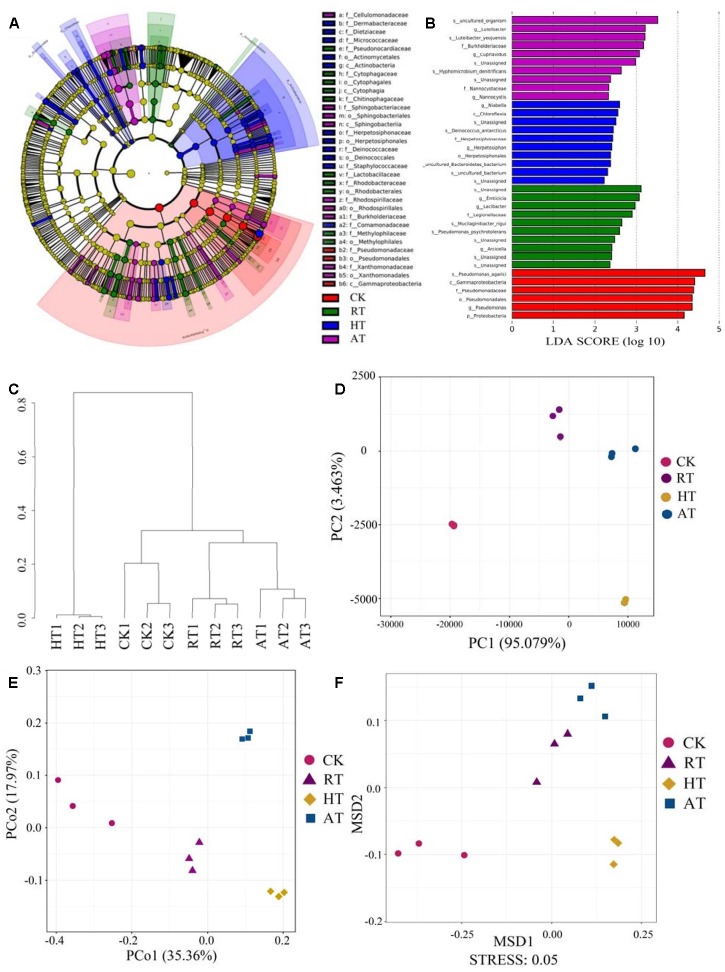
Beta diversity analysis of the exogenous bacteria in freeze-dried *Agaricus bisporus* under different storage conditions. **(A)** Cladogram generated from LEfSe analysis. **(B)** LDA scores of the differentially abundant taxa shown in **(A)**. Taxa enriched are indicated with LDA score (taxa with LDA score >2 and significance of a <0.05 determined by Wilcoxon signed-rank test). **(C)** Cluster analysis. **(D)** PCA. **(E)** PCoA. **(F)** NMDS.

The taxa difference with LDA scores greater than 2.0 were shown in Figure 3A,B. LEfSe and LDA were performed to identify the bacterial profile distinction, and LEfSe provided a global view of the bacterial composition changes in association with storage conditions. The taxa results demonstrated that the relative abundance of *Gammaproteobacteria* and *Pseudomonadaceae* were higher in CK samples than in treated samples. In addition, seven families and 15 genera were significantly higher in the HT group. Meanwhile, ten families and 12 genera were significantly higher in the RT group, while five families and 13 genera were significantly higher in the AT group. At the family levels, the relative abundance of *Cytophagaceae* and *Rhodobacteraceae* were enriched in RT samples, while *Sphingobacteriaceae* and *Rhodospirillaceae* were enriched in AT samples. Moreover, HT samples showed higher relative abundance in *Comamonadaceae*, *Dermabacteraceae*, and *Deinococcaceae*. Cluster analysis, PCA, principal coordinate analyses (PCoA) based on the unweighted UniFrac distances, and non-metric multidimensional scaling (NMDS) were shown in Figure 3C–F, respectively. The general taxonomic patterns were largely driven by differences in the abundance of major taxonomic groups. The cluster analysis indicated that samples were divided into two main clusters, the HT samples group and the CK, RT, and AT samples group. Moreover, the RT samples bacterial community was closer to that of AT samples. PCA and PCoA results showed the differences and similarities between the bacterial communities for all groups. The bacterial communities in the three replicated samples had a high similarity. In the results of NMDS analysis, CK samples and the treated samples were separated on MSD1, suggesting the significant difference among these samples.

### Bacterial Community at the Family Level

**FIGURE 4:**
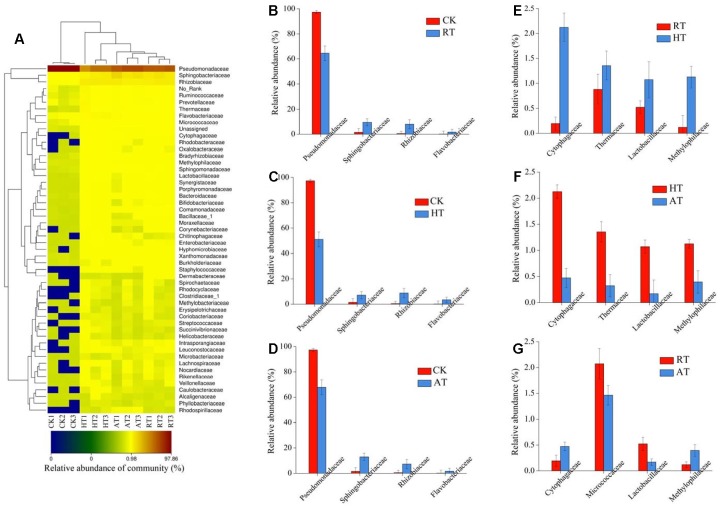
Heatmap **(A)** and significant difference between two groups revealed by Metastats at family level of the bacterial composition in freeze-dried *Agaricus bisporus* under different storage conditions (**B**: CK vs. RT, **C**: CK vs. HT, **D**: CK vs. AT, **E**: RT vs. HT, **F**: HT vs. AT, **G**: RT vs. AT).

Taxonomy heatmap reflects the actual similarities and differences in community composition of these samples. The bacterial community structure of freeze-dried *A. bisporus* was visualized using a heatmap by employing the relative abundance of each family. The findings were compared by using a hierarchical dendrogram (Figure 4A). The similarity between samples decreased from higher to lower taxonomic levels. At the family level, *Pseudomonadaceae*, *Sphingobacteriaceae*, and *Rhizobiaceae* were detected as the main family in all samples. The results showed that CK group exhibited the highest abundance of *Pseudomonadaceae*. Cluster analysis showed that bacterial communities in RT samples were clustered closer to AT samples, while the bacterial communities between CK samples and treated samples displayed a further relationship. Exogenous bacterial profiles of freeze-dried *A. bisporus* with significant difference between two groups were shown in Figure 4B–G. The relative abundance of *Sphingobacteriaceae*, *Rhizobiaceae* and *Flavobacteriaceae* was enriched in the treated samples compared to CK samples. The relative abundance of *Cytophagaceae*, *Thermaceae*, *Lactobacillaceae*, and *Methylophilaceae* in HT samples was significantly higher than that in RT and AT samples. In addition, the relative abundance of *Micrococcaceae* and *Lactobacillaceae* in RT samples was higher than that in AT samples.

### Bacterial Composition at the Genus Level

**FIGURE 5:**
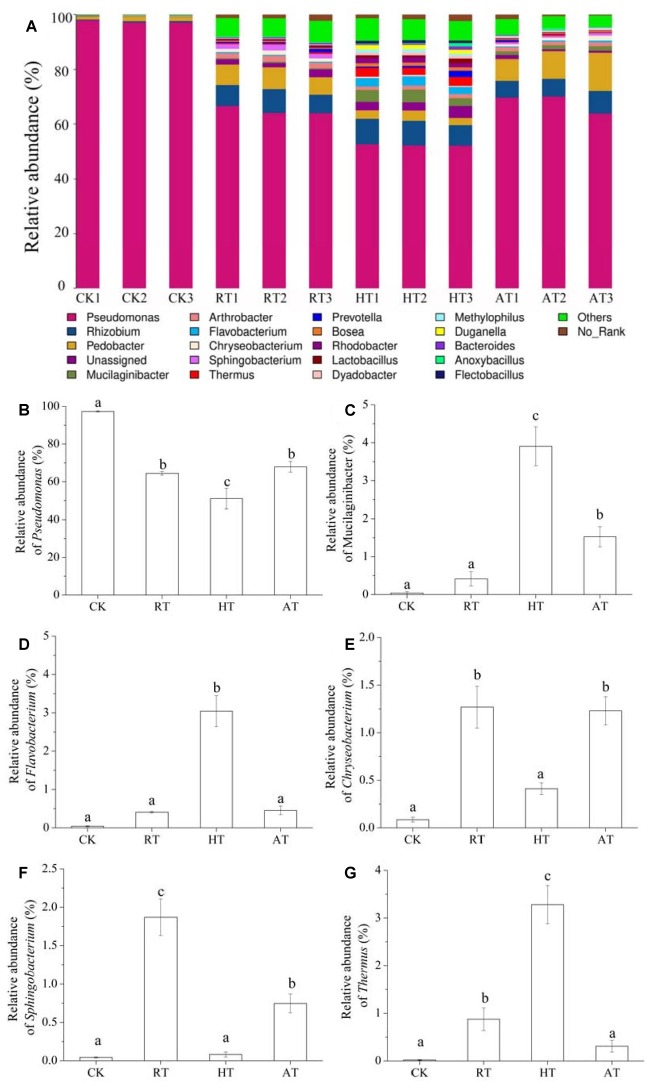
Bacterial composition at the genus level of freeze-dried Ag*aricus bisporus* under different storage conditions (**A**: stack column, **B**: relative abundance of *Pseudomonas*, **C**: relative abundance of *Mucilaginibacter*, **D**: relative abundance of *Flavobacterium*, **E**: relative abundance of *Chryseobacterium*, **F**: relative abundance of *Sphingobacterium*, **G**: relative abundance of *Thermus*).

Figure 5 showed the bacterial composition at the genus level in freeze-dried *A. bisporus*. Although different treated samples clearly harbored distinct bacterial communities, four groups had relatively similar bacterial community composition. The results showed that *Pseudomonas* was the main dominant genus in freeze-dried *A. bisporus*, followed by *Rhizobium* and *Pedobacter* genus. Additional genus were also present at even lower abundance. In addition, HT treatment significantly enhanced the relative abundance of *Mucilaginibacter*, *Flavobacterium*, and *Thermus*. It was noted that *Sphingobacterium* were enriched in RT samples, followed by AT samples. Moreover, the relative abundance of *Chryseobacterium* increased in RT and AT samples.

### GC-MS Analysis

**FIGURE 6:**
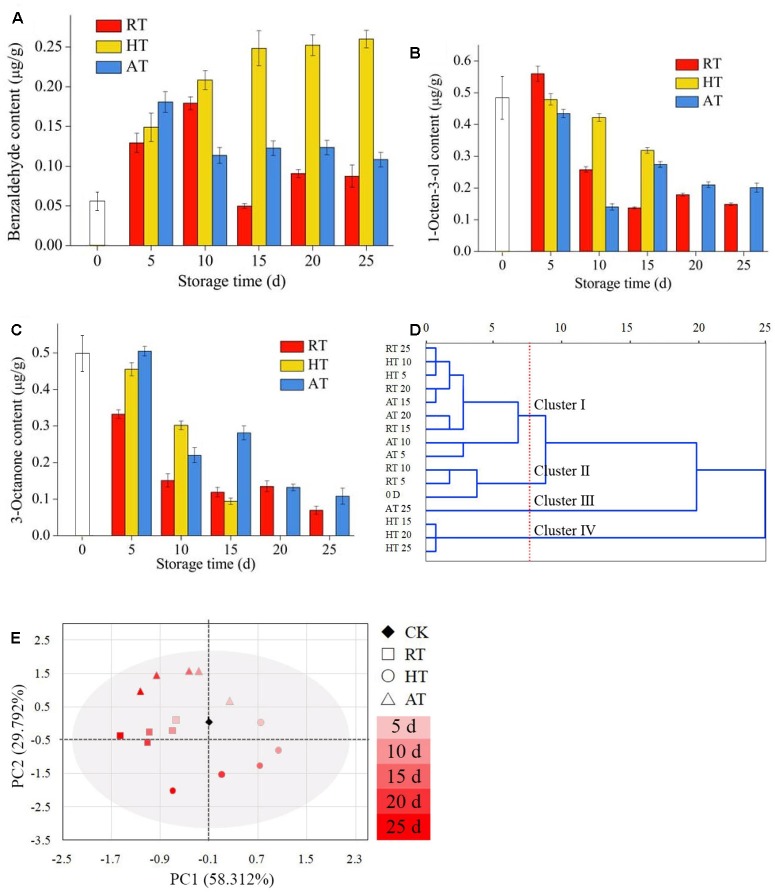
Volatile compounds in freeze-dried Ag*aricus bisporus* under different storage conditions (**A**: benzaldehyde content, **B**: 1-octen-3-ol content, **C**: 3-octanone content, **D**: cluster analysis, **E**: PCA).

Changes in volatile compounds of freeze-dried *A. bisporus* under the three storage conditions were monitored using SPME-GC-MS to provide the odor profile of each sample. A total of 35 different volatiles were detected by SPME-GC-MS and are presented in Table 1. Five kinds of representative flavor compounds were identified from freeze-dried *A. bisporus*, namely, aldehydes, ketones, alcohols, hydrocarbon and esters. Several volatile C8 compounds were identified as characteristic of freeze-dried *A. bisporus*. The main volatile compounds in freeze-dried *A. bisporus* included 3-octanone, 1-octen-3-ol, undecane, and 3-octanol. The content of aldehydes increased at the end of storage, while the content of ketones, alcohols and hydrocarbon decreased. It was noted that the content of esters in RT and AT samples increased, while that in HT samples decreased on day 25. Moreover, the contents of benzaldehyde, 1-octen-3-ol, and 3-octanone during the storage of 25 days were shown in Figure 6A–C, respectively. Compared to RT and AT samples, 1-octen-3-ol content in HT samples significantly decreased and was not detected on day 25. However, the benzaldehyde content in HT samples increased during storage. A cluster analysis was conducted to establish differences in the volatile compounds among samples according to squared Euclidean distance methods (Figure 6D). These clusters got closer with the Euclidean distance increase, which meant that there were some similarities between them. Generally, 16 samples were classified into two main groups within the 25 units distance. In the closer distances of four units, the examined populations were divided into four groups. For a better visualization, PCA analysis was performed in Figure 6E. The variance contribution rates for the first and second PC were 88.104%, hence the most odor information of samples could be used to identify the discrimination among freeze-dried *A. bisporus* under different storage conditions. The results indicated that these samples could be distinguished on the basis of their characteristic flavor during storage. Moreover, the value of PC1 decreased in samples during storage. The region of RT samples was close to the CK samples, which also showed similarities compared with the result in cluster analysis.

**Table 1:** Volatile compounds content in freeze-dried *Agaricus bisporus* on day 25 of storage under different conditions.

No	Category	Compounds name		Volatile compounds content (μg/g)			
				CK	RT	HT	AT
	Aldehydes						
1		Benzaldehyde		0.057 ± 0.012	0.087 ± 0.014	0.260 ± 0.011	0.108 ± 0.009
2		Octanal				0.044 ± 0.015	
3		Benzeneacetaldehyde				0.015 ± 0.003	
4		Decanal		0.007 ± 0.002	0.051 ± 0.010		0.011 ± 0.002
	Ketones						
5		3-Octanone		0.500 ± 0.129	0.069 ± 0.011		0.108 ± 0.022
6		2-Undecanone				0.013 ± 0.002	
7		5,9-Undecadien-2-one, 6,10-dimethyl-			0.021 ± 0.008	0.015 ± 0.005	
	Alcohols						
8		3-Octanol		0.164 ± 0.051	0.050 ± 0.013		0.037 ± 0.012
9		1-Octen-3-ol		0.486 ± 0.068	0.148 ± 0.015		0.201 ± 0.014
10		Benzyl alcohol		0.073 ± 0.019	0.052 ± 0.014		0.023 ± 0.013
11		2-Nonen-1-ol, (E)-		0.111 ± 0.018			0.013 ± 0.006
12		1-Octanol		0.058 ± 0.019	0.023 ± 0.008		
13		Nonanal		0.063 ± 0.012	0.088 ± 0.016	0.077 ± 0.028	0.076 ± 0.014
	Hydrocarbon						
14		Heptane, 2,2,4,6,6-pentamethyl-		0.136 ± 0.022		0.028 ± 0.009	
15		Decane, 3,7-dimethyl-				0.026 ± 0.007	
16		Undecane		0.309 ± 0.089	0.083 ± 0.016	0.024 ± 0.008	
17		Dodecane		0.099 ± 0.018	0.010 ± 0.003	0.015 ± 0.006	
18		Tetradecane, 4-methyl-				0.060 ± 0.013	0.033 ± 0.011
19		Tridecane		0.039 ± 0.017		0.030 ± 0.011	
20		Heptadecane				0.030 ± 0.007	
21		Octacosane				0.015 ± 0.003	
22		Undecane, 2,6-dimethyl-				0.029 ± 0.016	
23		Tridecane, 3-methyl-		0.043 ± 0.012		0.016 ± 0.007	
24		Tetradecane		0.087 ± 0.012	0.011 ± 0.002	0.026 ± 0.014	
25		Hentriacontane				0.026 ± 0.013	
26		Hexadecane		0.027 ± 0.013	0.009 ± 0.002		
27		1-Decene		0.060 ± 0.019	0.038 ± 0.012		0.021 ± 0.011
28		1-Dodecene		0.017 ± 0.004			
29		1-Tetradecene		0.029 ± 0.013	0.032 ± 0.010		
	Esters						
30		Hexanoic acid, methyl ester					0.028 ± 0.015
31		Octanoic acid, methyl ester		0.027 ± 0.09			
32		Tetradecanoic acid, 12-methyl-, methyl ester					0.012 ± 0.004
33		Decanoic acid, methyl ester		0.029 ± 0.013	0.043 ± 0.011		0.033 ± 0.012
34		Sulfurous acid, 2-propyl tetradecyl ester				0.012 ± 0.002	
35		Sulfurous acid, hexyl octyl ester			0.048 ± 0.011		
Total content of compounds				2.421 ± 0.571	0.864 ± 0.176	0.761 ± 0.181	0.749 ± 0.150

## Discussion

In the present study, we demonstrated the effect of storage condition on the exogenous bacterial composition and resultant volatile compounds changes. The results showed that the temperature and humidity have a significant effect on exogenous bacterial composition of freeze-dried *A. bisporus* during storage. Storage temperature and humidity are considered important factors for maintaining food quality by affecting the lag phase duration and the growth rate of bacterial populations (Doulgeraki et al., 2012). Total viable counts in HT samples increased and were higher than that of RT and AT samples during the storage for 25 days, suggesting that the storage conditions led to the bacterial populations changes. Compared to HT treatment, RT and AT treatments extended the lag phase of bacterial growth and slowed the propagation of bacteria. It was observed that these treatments enhanced the diversity and richness of the bacterial community of freeze-dried *A. bisporus* with the diversity indices increase including OTU numbers and Shannon index. This suggested that the increase of diversity were due to the specific bacteria groups changes induced by storage conditions (Tajima et al., 2007). Similar to the alpha diversity patterns, the concordance in beta diversity patterns indicated that the overall differences among the bacterial communities were significantly correlated with the composition differences of these communities (Fierer et al., 2013). The sequences from exogenous bacteria in freeze-dried *A. bisporus* were assigned to 12 phyla with the majority of reads belonging to the *Proteobacteria*, *Bacteroidetes*, and *Firmicutes*. In addition, *Proteobacteria*, *Bacteroidetes*, and *Firmicutes* are the dominant members of mushrooms (Venturini et al., 2011). The results proved that *Pseudomonas* was the main dominant genus in the samples (Godfrey et al., 2001). Most members of the *Pseudomonas* genus produce active proteinases and lipases, which give them advantages over possible competitors by adjustment of several enzymatic pathways (Chung et al., 2014). *Pseudomonas tolaasii* can live as a saprophytic or as a pathogenic microorganism, and can even infect and facilitate the damage of almost all species of mushrooms (Soler-Rivas et al., 1999). Moreover, tolaasin, produced only by *P. tolaasii* can lead to pitting and browning of *A. bisporus* (Abou-Zeid, 2012). Furthermore, *Pseudomonas agarici* is responsible for drippy gills symptoms but also for brown discoloration (Largeteau and Savoie, 2010). In addition, microbial symbionts have been proved to benefit their hosts through defense against pathogens and biosynthesis of essential nutrients (Aylward et al., 2014). *Pseudomonas* has substantial biosynthetic capacity which may affect the nutrition of *A. bisporus*, including amino-acid transport and metabolism, carbohydrate transport and metabolism, and inorganic ion transport and metabolism (Aylward et al., 2012; Pecchia et al., 2014). In addition, the relative abundance of *Mucilaginibacter*, *Flavobacterium* and *Thermus* in HT samples significantly increased. *Thermus* is a genus of thermophilic bacteria, and the biodiversity among thermophiles is largely determined by temperature (Lau et al., 2009). Moreover, *Chryseobacterium* was the other genera more dominant in AT samples, *Sphingobacterium* and *Chryseobacterium* were a few other genera more dominant in RT samples.

Freeze-dried *A. bisporus* has its own unique flavor, and some studies have been focused on its flavor analysis. However, flavors of freeze-dried *A. bisporus* can change and deteriorate during storage, and the olfactory impact may be a result of microbial development. The results showed that the volatile compounds identified in freeze-dried *A. bisporus* included aldehydes, ketones, alcohols, hydrocarbon and esters. It has been reported that *Pseudomonas* can produce volatile organic compounds, recognized as active odor molecules, which are possibly responsible for off-odor release during storage (Kim et al., 2016). Moreover, *Pseudomonas* is considered as the microorganism able to produce the larger number of aldehydes, such as benzaldehyde and benzeneacetaldehyde (Casaburi et al., 2015). It was noted that the benzaldehyde content had a significant positive correlation with the growth of Pseudomonads in HT samples (*p* < 0.05). Therefore, the growth of Pseudomonads in the storage at high temperature and humidity may induce the release of benzaldehyde which resulted in the odor deterioration in freeze-dried *A. bisporus*. Ketones in the freeze-dried *A. bisporus* increased during storage, which are the important volatile metabolite of *Pseudomonas* (Hilton and Cain, 1990; Filipiak et al., 2012). Kumar et al. (2011) reported that a ‘fruity’ or ‘cheesy’ odor is mostly produced by *Chryseobacterium*. Thus, the growth of *Chryseobacterium* abundance in RT and AT samples may induce the esters production which imparts the fruity aroma. *Flavobacteria* could produce the volatile compounds included alcohols, ketones, aldehydes, esters, and the resultant odors have been described as fishy, foul (Mulla et al., 2018). The relative abundance of *Flavobacterium* in HT samples significantly increased. This may lead to odor deterioration of freeze-dried *A. bisporus* and even low market acceptance. However, volatile compounds changes induced by the specific strain remained unclear. Thus, further studies are needed in order to monitor the growth of strains during storage as a comparison of volatile compounds.

## Conclusion

In the present study, the exogenous bacterial composition and volatile compounds of freeze-dried *A. bisporus* during storage were investigated. Lower water activity in RT and AT samples extended the lag phase of bacterial growth and slowed their propagation, while the total bacterial colonies significantly increased in HT samples owing to the high humidity. This study showed that bacterial diversity in freeze-dried *A. bisporus* increased during storage, and HT samples had the highest diversity index among these groups. Moreover, beta diversity analysis of the bacterial communities suggested that the exogenous bacteria in freeze-dried *A. bisporus* exhibit unique composition during storage. Nevertheless, the dominant bacteria population in freeze-dried *A. bisporus* was *Proteobacteria*, *Bacteroidetes*, and *Firmicutes*. However, at the genus level, the composition was dominated by *Pseudomonas*, followed by *Rhizobium* and *Pedobacter*. The analysis showed that *Mucilaginibacter*, *Flavobacterium*, and *Thermus* were a few other genera more dominant in HT samples, whereas, *Sphingobacterium* and *Chryseobacterium* were a few other genera more dominant in RT samples, and *Chryseobacterium* were the other more dominant in AT samples. Furthermore, the increasing benzaldehyde content in HT samples may have been induced by the growth of *Pseudomonads*. Moreover, the increasing relative abundance of *Chryseobacterium* in RT and AT samples may have induced the esters production. The results provided comprehensive information about the biodiversity and the structure of bacterial community in freeze-dried *A. bisporus* during storage. Furthermore, the study provides significant information for the safety and quality monitor for freeze-dried *A. bisporus*.

## Author Contributions

QH and FP contributed to the conception and design of the study. LW organized the database and performed the statistical analysis. WY and MM wrote sections of the manuscript. All authors contributed to manuscript revision, read and approved the submitted version.

## Conflict of Interest Statement

The authors declare that the research was conducted in the absence of any commercial or financial relationships that could be construed as a potential conflict of interest.
